# Nutritional Composition, Volatile Profiles, and Biological Evaluation of Honeys from *Melipona interrupta* and *Melipona seminigra* from Amazonas State, Brazil

**DOI:** 10.3390/plants14142106

**Published:** 2025-07-09

**Authors:** Emilly J. S. P. de Lima, Carlos V. A. da Silva, Fernanda A. S. Rocha, Aline de M. Rodrigues, Samuel C. Costa, Rebeca S. França, Raiana S. Gurgel, Bárbara N. Batista, Patrícia M. Albuquerque, Waldireny R. Gomes, Hector H. F. Koolen, Giovana A. Bataglion

**Affiliations:** 1 Metabolomics and Mass Spectrometry Research Group, University of the State of Amazonas (UEA), Manaus 69065-001, Brazil; emillyjulianasales@gmail.com (E.J.S.P.d.L.); cv25066@gmail.com (C.V.A.d.S.); feradrielle@gmail.com (F.A.S.R.); admr.mbt24@uea.edu.br (A.d.M.R.); 2 Department of Chemistry, Federal University of Amazonas (UFAM), Manaus 69077-000, Brazil; costa.samuelcarvalho@gmail.com (S.C.C.); rdss.franca@gmail.com (R.S.F.); 3 Laboratory of Chemistry Applied to Technology, School of Technology, University of the State of Amazonas (UEA), Manaus 69050-020, Brazil; raianagurgel@hotmail.com (R.S.G.); barbara_sing@hotmail.com (B.N.B.); palbuquerque@uea.edu.br (P.M.A.); 4 Faculty of Pharmacy, Federal University of Amazonas (UFAM), Manaus 69077-000, Brazil; waldirenyrocha@ufam.edu.br

**Keywords:** bee product, volatilome, antioxidant, nutritional, chemical composition

## Abstract

Honey is a natural product produced by bees from the nectar of plants and has been widely used as a sweetener for centuries. In addition to its traditional use, it is also employed for other purposes due to its biological and nutraceutical properties. Although honey production is mostly associated with bees of the genus *Apis*, species from other genera, such as *Melipona*, also produce it, albeit on a smaller scale. The honey produced by these two genera shows significant differences in its composition. Moreover, distinct geographical localizations, which, consequently, have different flora, guide the chemical compositions of these samples. Regarding the Amazon region, the amount of knowledge about the honey samples from *Melipona* species is still scarce. In this context, the present study aimed to characterize the volatile compositions of honey from *Melipona interrupta* and *Melipona seminigra*, as well as from the floral sources available, in addition to evaluating their nutritional aspects, antioxidant activity, and antibacterial activity. The analysis of chemical composition was performed using gas chromatography coupled to mass spectrometry (GC-MS). Antioxidant activity was determined by DPPH and ABTS assays, while antimicrobial activity was tested against *Escherichia coli*, *Staphylococcus aureus*, *Klebsiella pneumoniae*, *Pseudomonas aeruginosa*, *Proteus mirabilis*, *Staphylococcus epidermidis*, *Enterococcus faecalis*, *Salmonella enterica*, *Serratia marcescens*, *Bacillus subtilis*, *Candida albicans*, *Candida tropicalis*, and *Candida parapsilosis.* The results allowed the identification of volatiles present in the honey and floral sources. The samples displayed moderate antioxidant activity and slightly antibacterial activity (MIC) of 75 μg/mL against two bacterial strains tested, demonstrating potential antimicrobial activity.

## 1. Introduction

Honey is recognized as a sweet and viscous food, predominantly synthesized by stinging bee species, with *Apis mellifera* being the most widely used in commercial production. It can be classified as either monofloral or multifloral [1,2]. On a smaller scale, some stingless bee species, initially cultivated by indigenous peoples, also produce honey [3].

Stingless bee honey is a food product made from the nectar of flowers or from the secretions of living parts of plants. The bees collect these substances, process them, and mix them with their own enzymes, storing the final product in the colony pots, where it undergoes a maturation process [4]. In Brazil, around 259 species of stingless bees are found, distributed across 28 genera [5]. The state of Amazonas stands out as a region that hosts a large portion of these species, presenting the greatest diversity of meliponines in the country [6]. The characteristics of honey produced by stingless bees have contributed to its increasing recognition as a potential functional food since its bioactive compounds have been associated with various nutraceutical and medicinal effects [7]. In vitro studies confirm the properties exhibited by this product, including antimicrobial [8], anti-inflammatory, antioxidant [9,10], antitumoral [10], and antidiabetic [11] activities.

Furthermore, in vivo experimental studies have observed an important protective role against chronic inflammation caused by lipopolysaccharides (LPS), improving both inflammation and oxidative stress [12]. Honey produced by *Melipona subnitida* showed hypolipidemic and antioxidant effects, as well as protective effects against lesions caused by dyslipidemia in colonic epithelial cells in rats [13]. Additionally, assays confirmed an improvement in depressive behavior, a reduction in weight and body mass index, as well as improvements in lipid profile, leptin and insulin levels, HOMA-β, and glucose and insulin tolerance in obese rats [14]. Honey produced by species of this genus also demonstrated the ability to reduce metabolic endotoxemia by modulating the gut microbiota to prevent glucose intolerance [15]. These beneficial effects may be directly related to its chemical composition, which goes beyond sugars.

Although the chemical composition of honey is predominantly made up of sugars, its chemical profile is quite diverse, containing proteins, carbohydrates, amino acids, vitamins, minerals, fatty acids, enzymes, phenolic compounds, and terpenes, which may be related to the properties it exhibits [1,16]. Regarding the latter, those belonging to the monoterpene and sesquiterpene representatives form part of the class of volatile organic compounds (VOCs) in addition to other components derived from fatty acid degradation, all commonly found in honey samples. These comprise a group of lipophilic natural products with low molecular weight and high vapor pressure at room temperature. There are numerous varied compounds widely distributed in the flora and incorporated/modified in the honey samples [17,18].

These compounds can mediate intra- and interspecific relationships, often being involved in the attraction of pollinators. In plants, flowers are generally the main producers of the most significant quantities and varieties of these metabolites [19,20]. The floral volatile profile is specific and varies depending on the type of pollinator. However, some flowers preferentially attract certain bee species, while others are more generalist [21,22]. Many floral VOCs exhibit antimicrobial activity and can also repel pollinators, directing them to parts that have not yet been pollinated. Furthermore, these compounds play a fundamental role in the flavor of honey since, depending on the type of honey, it presents a characteristic taste of the plant due to the presence of volatile organic compounds in the nectar [23,24].

It is worth noting that the chemical composition of honey is influenced by several factors, such as the characteristics of the plants from which the bees collect nectar, as well as the characteristics of the region, the time of collection, and the species of the bees producing it. Therefore, variations in chemical composition related to the region are frequently found in its chemical profile [25,26].

The Amazon region concentrates a large portion of the stingless bee species found in Brazil, totaling approximately 197 species, of which 128 occur in the state of Amazonas [5]. Considering that many of these species are little known and underexplored, there has been growing interest in investigating the potential of the products synthesized by them, particularly those of the *Melipona* genus. This genus has been widely used by meliponiculturists in the region, comprising around 40 species. Of this total, according to the review, only 28 species have any type of chemical or biological study [27].

Therefore, the present study investigated the volatile chemical composition of honey produced by *M. interrupta* (MIH) and *M. seminigra* (MSH), as well as of the floral sources near the hives located in the municipality of Iranduba (Amazonas state, Brazil). Additionally, analyses of the antibacterial potential, antioxidant activity, and nutritional profile of the honeys were also conducted. The study aims to contribute relevant information about the honey from these species, highlighting their nutraceutical and biological properties to promote their appreciation and encourage their use.

## 2. Results

### 2.1. Nutritional Facts

The present study evaluated the moisture, ash, protein, lipid, carbohydrate, reducing sugars, non-reducing sugars, and total caloric value of the honey samples (detailed results in Table 1).

The honey MSH presented a higher moisture content (27.11 g/100 g) compared to MIH (20.11 g/100 g).

The ash content was also higher in MSH (0.31 g/100 g), which was double the amount found in MIH (0.15 g/100 g). Regarding protein content, MIH showed a slight difference compared to MSH. The values found were 0.19 and 0.16 g/100 g, respectively. There are no reference values for this parameter; however, studies indicate that the protein content found in stingless bee honey is generally low [28].

The lipid content found in the two samples was very similar: 0.20 g/100 g in MIH and 0.19 g/100 g in MSH.

The *M. interrupta* honey showed a carbohydrate content of 79.35 g/100 g, higher when compared to *M. seminigra*, which presented a value of 72.24 g/100 g. Found values ranging from 48.59% to 69.36% in stingless bee honey samples [29], slightly lower than those found in the present study.

The percentage of reducing sugars showed a difference between the analyzed samples: 76.95 g/100 g in MIH and 68.75 g/100 g in MSH. Therefore, MSH is more in line with the established limit.

Regarding non-reducing sugars, MSH presented a value twice as high as that of MIH, with values of 3.14 g/100 g (3.1%) and 1.43 g/100 g (1.4%), respectively. Therefore, the samples analyzed here are within the established standard. Values ranging from 7.2% to 8.5% have been reported for honey from *M. fasciculata* [30]. The total caloric value was higher in MIH (305.71 kcal/100 g) than in MSH (278.34 kcal/100 g). The reducing sugars influence the energy value of honey, characterizing it as an immediate source of energy [31].

### 2.2. Radical Scavenging Activity

The two radical scavenging activities of MIH and MSH were determined using the DPPH and ABTS methods. The values obtained are listed in Table 2. The inhibition percentage against the DPPH radical was (49.98% ± 0.75) and (46.14% ± 1.83), respectively. Although the difference is small, honey from *M. interrupta* showed slightly higher inhibition capacity compared to that from *M. seminigra*.

For the ABTS radical, the percentages found were (58.73% ± 2.33) for MIH and (60.17% ± 1.68) for MSH. The difference between the percentages was also subtle. These slight differences suggest variations in the composition of bioactive compounds between the species.

The IC_50_ values obtained in the DPPH test were (987.7 μg/mL ± 0.02) for MIH and (953.3 μg/mL ± 0.01) for MSH, indicating that the latter exhibited higher antioxidant activity in this assay. In the ABTS test, the IC_50_ values were (65.49 μg/mL ± 0.02) for MIH and (67.73 μg/mL ± 0.01) for MSH, showing greater antioxidant activity for honey from MIH.

### 2.3. Antimicrobial Activity

The honey samples were tested against the microorganisms *Escherichia coli* (CCCD-E005), *Staphylococcus aureus* (CCCD-S009), *Klebsiella pneumoniae* (CCCD-K003), *Pseudomonas aeruginosa* (CCCD-P004), *Proteus mirabilis* (CCCD-P001), *Staphylococcus epidermidis* (CCCD-S010), *Enterococcus faecalis* (CCCD-E002), *Salmonella enterica* (CCCD-S003), *Serratia marcescens* (CCCD-S005), *Bacillus subtilis* (CCCD-B005), *Candida albicans* (CCCD-CC001), *Candida tropicalis* (CCCD-CC002), and *Candida parapsilosis* (CCCD-CC004).

Just two Gram-positive bacterial strains, *E. faecalis* and *B. subtilis*, showed a Minimum Inhibitory Concentration (MIC) of 75 μg/mL. The results showed no differences between the honey species and the bacterial strains and are listed in Table 3. The antibacterial activity of honey produced by the *Melipona* species has already been previously reported in the literature [32].

### 2.4. Volatile Profiles

**Figure 1:**
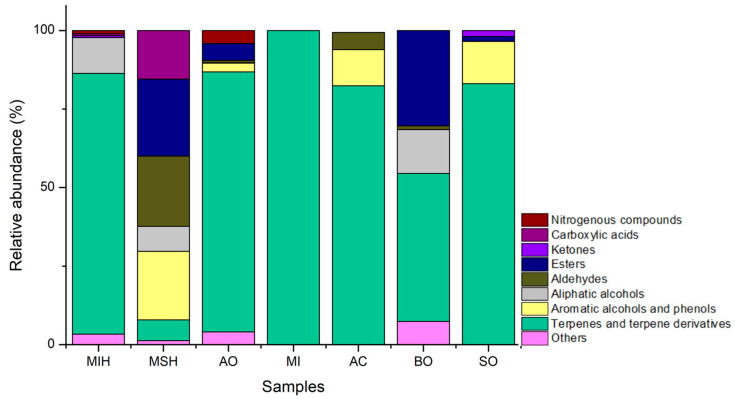
Classes of chemical compounds found in the analyzed samples. **MIH**: honey of *M. interrupta*; **MSH**: honey of *M. seminigra*; **AO**: flowers of *Anacardium occidentale*; **MI**: flowers of *Mangifera indica*; **AC**: flowers of *Averrhoa carambola*; **BO**: flowers of *Bougainville* sp.; **SO**: flowers of *Senna occidentalis*.

The analysis by GC-MS allowed the identification of volatile compounds among the stingless bee honey samples and their floral sources, distributed across the classes of terpenes, alcohols, esters, ketones, and aldehydes (Table 4). Among these, terpenes were the most abundant, followed by esters and alcohols (Figure 1). Alcohols represent a large and important class of compounds present in honey [33] (The chromatograms corresponding to the analyzed samples are available in the Supplementary Material (Figures S1–S7)).

The analysis of the volatile chemical composition of the honey samples revealed the presence of four compounds in common among the investigated species: hotrienol 42% in MIH and 3% in MSH; linalool in MIH and MSH (2.6% and 1.6% in MIH and MSH, respectively); decanal (0.6% and 0.02% in MIH and MSH, respectively); and nonanoic acid (0.6% in MIH, and 2.4% in MSH). In addition to these shared constituents, it was observed that the samples derived from *M. seminigra* exhibited a chemical profile with more evenly distributed classes among carboxylic acids, esters, aldehydes, alcohols, and terpenes when compared to *M. interrupta*, which presented a composition predominantly rich in terpenes. Terpenes represented the predominant class of secondary metabolites in all floral samples analyzed. Quantitatively, a significant variation was observed in the number of identified compounds: the *Anacardium occidentale* (AO, 31 compounds) and *Senna occidentalis* (SO, 24 compounds) showed greater diversity compared to *Mangifera indica* (MI, 11 compounds), *Averrhoa carambola* (AC, 15 compounds), and *Bougainville* sp. (BO, 18 compounds).

Furthermore, the monoterpene linalool was common in almost all analyzed samples: in MIH (2.6%), MSH (1.6%), AO (0.1%), MI (3.8%), AC (1.2%), and BO (1.6%). Another terpene that was also common in several samples was β-ocimene, which was present in MIH (0.4%), AC (50.9%), BO (8.8%), and SO (40.7%). In addition, terpinolene, found in MSH (1.1%), was also present in AO (1%) and MI (11.5%).

Phenethyl alcohol was one of the major constituents in MSH (12.3%) and SO (13.5%) and was also detected in AO (1.3%) and AC (1.6%). The compound 1-nonanol, found in MIH (8.1%), was also present in AO (0.1%) and AC (1.8%). Decanal was detected in both honey samples, MIH (0.6%) and MSH (0.02%), as well as in BO (0.3%). Also, 3-hexen-1-ol was present in similar proportions in AC (7.4%) and BO (7.1%). These samples also shared the presence of (*E*)-linalool oxide, found at 0.41% in AC and 14.3% in BO. The compound cosmene was common to the AC, BO, and SO samples. Cosmene was found at 1.8% in AC, 4.8% in BO, and 5.2% in SO.

In AO, the analysis revealed β-caryophyllene (26.2%) as the major compound, followed by α-copaene (6.7%), 3-carene (6.1%), α-caryophyllene (5.1%), δ-cadinene (5%), γ-cadinene (4.7%), γ-gurjunene (4.7%), and γ-muurolene (4%). Compounds such as caryophyllene, cadinene, and muurolene are frequently found in *Melipona* honey, indicating a direct transfer of floral volatiles to the final product [34].

In MI, the main compounds identified were 3-carene (20.9%), *α*-pinene (19%), *β*-myrcene (10.4%), terpinolene (11.4%), β-pinene (8.6%), D-limonene (6.9%), 4,8-dimethylnone-1,3,7-triene (5.7%), linalool (3.8%), and sabinene (2.9%). The presence of these monoterpenes and sesquiterpenes, commonly associated with tropical floral profiles, is also observed in stingless bee honey [35].

In AC, the most prominent compounds were (*E*)-β-ocimene (50.9%), *α*-farnesene (16.6%), 3-hexen-1-ol (*Z*) (7.4%), nonanal (5.4%), (*Z*)-β-Ocimene (3.1%), lavandulol (2.4%), cosmene (1.8%), 1-nonanol (1.8%), and phenethyl alcohol (1.6%). Compounds such as (*E*)-β-ocimene and phenethyl alcohol are widely reported in stingless bee honey and are associated with floral attraction and the antioxidant bioactivity of these products [27].

The volatile composition of BO was predominantly composed of 3-hexenyl 2-methylbutyrate (26.3%), (*E*)-linalool oxide (14.3%), (*E*)-β-ocimene (8.8%) 3-hexen-1-ol (*Z*) (7.1%), 4-penten-1-ol (6.8%), cosmene (4.8%), hexyl isobutyrate (3.4%), and linalool (1.6%). The similarity between floral volatiles and compounds identified in stingless bee honey highlights the role of this plant in the botanical origin of certain tropical honeys [36].

Finally, in *Senna occidentalis*, the identified compounds were (*E*)-β-ocimene (40.7%), phenethyl alcohol (13.5%), β-caryophyllene (12.3%), 3*E*,7*E*-4,8,12-trimethyltrideca-1,3,7,11-tetraene (6.8%), cosmene (5.2%), (*neo*-alloocimene (7.5%), *γ*-muurolene (3.2%), α,β-dihydro-β-ionone (1.9%), and α-copaene (1.4%). (*E*)-β-Ocimene is widely recognized for its role in bee attraction [37], while phenethyl alcohol and β-caryophyllene are compounds present in honeycombs of *Apis cerana* and *Apis mellifera*, and may also be present in *Melipona* honey depending on floral origin [38].

## 3. Discussion

### 3.1. Nutritional Aspects

In Brazil, there is currently no specific legislation that establishes physicochemical standards for stingless bee honey. Regulatory parameters have been defined only for *Apis mellifera* honey [39].

Honey is a complex mixture in which water constitutes a significant component that influences various physical properties, including viscosity and crystallization. Moisture content also plays a key role in determining characteristics such as color, flavor, specific gravity, solubility, and shelf life [33]. Moisture content is considered an indicator of honey maturity and can be directly influenced by factors such as the floral nectar source and regional climatic conditions, particularly relative humidity [40]. In the present study, moisture content in the analyzed samples was 20.11% for *Melipona interrupta* and 27.30% for *Melipona seminigra*. The moisture of samples exceeded the recommended limit of 20 g/100 g established by the Codex Alimentarius Committee [28] for *Apis mellifera*. However, the honey from *M. interrupta* showed a value closer to the established legal limit. These elevated values may be attributed to the environmental conditions at the collection site, as the municipality of Iranduba commonly exhibits relative humidity levels exceeding 80% (INMET). Comparable results were reported for *Melipona* spp. honey from the state of Pará, with moisture content ranging from 20.70% to 25.00% [41].

Similarly, observed values ranged from 19.23% to 31.82% in honey produced by other species within the same genus [42]. Supporting evidence is also provided, which found moisture contents of 27.2% and 27.0% in honey produced by *M. fasciculata* and *M. subnitida*, respectively, from the semi-arid region of northeastern Brazil [31]. Recent investigations have shown significant variation in honey moisture content among different stingless bee species, even when exposed to similar climatic conditions. In southern Brazil, for instance, *M. bicolor* honey exhibited moisture contents ranging from 51.3% to 54.3%, *M. marginata* from 48.5% to 53.3%, and *M. quadrifasciata* from 53.5% to 58.3% [43].

In contrast, analyses of honey samples produced in the state of Santa Catarina state (Brazil) revealed considerably higher moisture contents, ranging from 58.97% to 71.00% in *Melipona quadrifasciata*, 65.83% to 68.90% in *M. mondury*, 61.00% in *M. bicolor*, and 60.64% in *M. marginata* [9]. These differences may be attributed to various factors, including species-specific physiological characteristics and regional floral diversity.

The ash content of the samples analyzed was 0.15% for MIH and 0.31% for MSH; the committee established a maximum value of 0.6 g/100 g. In this case, both samples presented values well below the maximum limit. This parameter serves as an indicator of honey purity, as it reflects the mineral content, which may be influenced by environmental pollution and the geographical origin of the honey [44]. The type of soil in which the nectar-producing flowers grow also affects ash levels. Studies have shown that the average ash content in honey is approximately 0.17% (*w*/*w*), with reported values ranging from 0.02% to 1.03% (*w*/*w*), consistent with the values observed in this study [45]. Similar ranges were reported for honey samples produced and commercialized by *Melipona* spp. in the central Amazon region (Brazil), which varied from 0.02% to 0.33% [42]. In the same region, reported values were between 0.13% and 0.20% [41]. For honey produced by *M. subnitida*, ash content ranged from 0.03% to 0.20% [39]. In honey produced by *Melipona* species in temperate regions of southern Brazil, reported ash values included 0.07% for *M. bicolor*, 0.05% to 0.08% for *M. marginata*, and 0.05% to 0.28% for *M. quadrifasciata* [43].

The protein content in the honey samples analyzed was 0.16% for MSH and 0.19% for MIH, which falls within the typical range for honey [33,44]. Although knowledge about the protein characteristics of stingless bee honey remains limited, this parameter is relevant for detecting product adulteration. In samples of honey produced by *M. seminigra* from various locations in the state of Amazonas, protein content ranged from 0.08% to 0.30%, a range that encompasses the values reported in the present study. Furthermore, when comparing the results with those reported for other species within the *Melipona* genus, a noticeable variation in protein content can be observed. For instance, evaluated honey produced by different *Melipona* species in the state of Rio Grande do Norte and reported protein contents of 1.8% for *M. scutellaris*, 0.9% for *M. subnitida*, 0.1% for *M. compressipes*, 0.3% for *M. quinquefasciata*, and 0.2% for *M. quadrifasciata* [45].

The lipid content observed in the honey samples analyzed was 0.19% for MSH and 0.20% for MIH; the Codex Alimentarius does not establish reference values for this parameter. When evaluating lipid content in honey from *M. seminigra* and *M. compressipes* collected in the city of Manaus (Amazonas state, Brazil), reported values of 0.24% and 0.16%, respectively [46]. Similarly, lipid contents were 0.12% for *M. compressipes* and 0.18% for *M. rufiventris paraensis* in honey from the same region. In Itacoatiara municipality (Amazonas state, Brazil), the values reported were 0.30% for *M. seminigra* and 0.09% for *M. compressipes* [47].

The total carbohydrate content of the honey samples analyzed was 72.24% for MSH and 79.35% for MIH; for this parameter, reference values are also not established by the committee. Previous studies on honey from *M. seminigra* and *M. compressipes* collected in the municipalities of Manacapuru and Itacoatiara (Amazonas state, Brazil) reported average carbohydrate contents of 61% and 60%, respectively [48]. Additionally, in the state of Pará (Brazil), carbohydrate levels were found to be 67.36% for *M. fasciculata* and 65.21% for *M. flavolineata* [49]. In the state of Paraná (Brazil), reported values were 69% for *M. bicolor*, 68% for *M. marginata*, 72% for *M. quadrifasciata*, and 67% for both *M. scutellaris* and *M. seminigra* [50]. It is worth noting that these variations may be attributed to factors such as botanical origin, environmental conditions, and honey processing by bees, all of which directly influence the final composition of the product. According to [33], monosaccharides—primarily glucose and fructose—constitute approximately 75% of the total sugars in honey, while disaccharides account for 10–15%, with traces of other sugars also detected. [26] reported that the average concentration of reducing sugars in honey is approximately 76.65%, a value consistent with the findings of the present study. Similarly, when analyzing honey produced by *M. subnitida*, reported reducing sugar contents ranging from 50.50% to 72.77% [39]. The Codex Alimentarius [28] states that these values should be no higher than 65 g/100 g.

The total caloric content was calculated as the sum of energy contributions from proteins, lipids, and carbohydrates. Among the samples analyzed, MIH presented the highest caloric value (305 kcal), followed by MSH (278 kcal). These values align with previous studies, which reported similar energy contents in other stingless bee honeys. For example, *M. rufiventris paraensis* honey showed a caloric value of 305 kcal, while honey from *M. compressipes* ranged between 270 and 296 kcal. For *M. seminigra*, the reported caloric value was 293 kcal [48].

### 3.2. Radical Scavenging Activity

When evaluating the *radical scavenging activity* of the honey samples, it was generally observed that the samples exhibited greater sensitivity to the ABTS radical, corroborating the findings of [51], which reported higher antiradical activity in the ABTS assay. Similar results were also reported, which found that the ABTS radical inhibition potential was significantly higher—up to four times greater—than that observed in the DPPH assay [40]. This difference may be attributed to factors such as the composition and complexity of the honey samples, as well as their interactions with free radicals. Moreover, ABTS is more soluble and compatible with both aqueous and organic solvents, and its reactivity spans a wide pH range, which may contribute to the higher sensitivity of this assay [52]. Antioxidant activity has already been confirmed in other species of the *Melipona* genus. The IC_50_ values obtained for *M. interrupta* and *M. seminigra* (987.741 μg/mL and 953.313 μg/mL by DPPH; 65.491 μg/mL and 67.735 μg/mL by ABTS) in the present study indicate a relatively lower antioxidant activity when compared to other *Melipona* species. Nevertheless, while the DPPH results are considerably higher than those reported for species such as *M. subnitida* (IC_50_ ranging from 14.54 to 212.28 μg/mL) and *M. marginata* (1.8 mg GAE/100 g) [9,53], the ABTS values for *M. interrupta* and *M. seminigra* are more consistent with previously published data.

For example, honey produced by *M. subnitida* in the state of Paraíba (Brazil) showed ABTS IC_50_ values ranging from 6.1 to 9.7 mg/mL [39], and extracts from Brazilian honey ranged from 30.88 to 137.79 mmol TE/100 mg in ABTS-based assays [54]. In addition, honey from *Melipona* sp. in Pará state registered antioxidant capacities of 1.15 μM TROLOX/g and 1.06 μM TROLOX/g [41]. These findings suggest that although *M. interrupta* and *M. seminigra* honey exhibit lower antioxidant activity by the DPPH method, their ABTS results are more aligned with values typically observed in the *Melipona* genus. Such discrepancies between the methods may be due to variations in honey chemical composition or differences in assay sensitivity. Overall, the results underscore the diversity of antioxidant properties among *Melipona* species and highlight the need for further studies to better understand the factors influencing such variability.

### 3.3. Antibacterial Activity

The honey samples were also tested against various microorganisms, and two Gram-positive bacterial strains, *E. faecalis* and *B. subtilis*, were susceptible. (Table 3). The antibacterial activity of honey produced by *Melipona* species has previously been reported in the literature, with activity against *E. faecalis,* the CIM observed in honey from *M. grandis* (62.50 µg/mL), *M. flavolineata* (31.20 µg/mL), and *M. seminigra* (25 µg/mL) [32].

This same bacterial strain (*E. faecalis*) was also found to be susceptible to honey produced by *M. baccaerri* [55], as well as by *M. seminigra* (55 µg/mL) and *M. compressipes* (75 µg/mL) [56]. Strains of *B. subtilis* have also shown susceptibility to honey produced by other stingless bee species from the genera *Heterotrigona*, *Tetrigona*, and *Lepidotrigona* [57].

Honey presents a complex combination of physical, chemical, and biological factors that, acting synergistically, confer its natural antimicrobial properties. Among these factors are low water activity, high osmotic pressure, acidic pH, a glucose oxidase system with consequent hydrogen peroxide production, a high carbon-to-nitrogen ratio, low redox potential associated with elevated levels of reducing sugars, low protein content, as well as the presence of phytochemical components and volatile substances [58,59].

These variables, collectively, may be responsible for the antibacterial effects observed in the analyzed honey samples. Previous studies have demonstrated that secondary metabolites, such as phenols, flavonoids, and organic acids, along with the physicochemical factors of honey, play a relevant role in its antimicrobial activity [60].

Therefore, it is suggested that the antibacterial action of the evaluated honey results from the complex interaction of various bioactive compounds.

### 3.4. Volatile Chemical Composition

The volatile chemical composition of flowers has great ecological importance, as it plays a fundamental role in attracting pollinators, particularly stingless bees of the *Melipona* genus. This composition also influences the aromatic and functional profile of the resulting honey. Investigating floral volatile compounds provides a better understanding of the botanical origin of honey and its chemical variability.

The analysis of the chemical composition revealed that terpenes were the most abundant class of compounds. A study on the volatile profile of honey produced by stingless bees of the *Melipona* genus indicated that acyclic monoterpenes, such as linalool derivatives, are the most prevalent compounds, regardless of geographical or entomological origin [59].

Similar results were reported by [61], who found that terpenes were the dominant class in the volatile composition of *M. scutellaris* honey, followed by esters and alcohols. Likewise, when analyzing the volatile profile of monofloral *Melipona* honey, terpenes were also identified as the predominant class, followed by esters [62].

The honey produced by our sample of *M. interrupta* had hotrienol as the major compound, accounting for 42.4% of the volatile composition. This compound is characterized by a fresh, floral, and fruity aroma and has a low odor threshold (0.11 ppm) [23].

In addition to hotrienol, other compounds were identified in significant proportions, such as (*Z*)-linalool oxide (9.2%), which is characterized by a fresh, sweet, and floral aroma with an odor threshold of 0.006 ppm [23]. 1-Nonanol (8.08%) presents a slightly floral and fruity aroma and has an odor threshold of 0.0009 ppm [61].

*α*-Terpineol (7.3%) was also detected, known for its green and floral scent and an odor threshold of 0.046 ppm [23]. Nerol oxide (7.2%) is another notable compound, described as having a floral, green, and sweet aroma [61].

Among the other major volatiles, *p*-mentha-1,5-dien-8-ol (4.9%) and linalool (2.6%) were identified, with the latter exhibiting a fresh and floral scent and an odor threshold of 0.006 ppm [23]. (*Z*)-Jasmone (2.5%) and 2-ethyl-1-hexanol (2.2%) were also present, with the latter described as having a characteristic grassy note [62]. Lastly, rose oxide (2.1%) was identified, known for its floral and green aroma [63].

On the other hand, the honey produced by *Melipona seminigra* (MSH) had benzaldehyde as the major volatile compound, representing 14.7% of the composition. This compound is typically associated with a pleasant honey-like, almond, sweet, and fruity aroma, with an odor threshold of 0.0042 ppm [63,64].

Other significant compounds included phenethyl alcohol (12.3%), characterized by a honey-like, spicy, rose, and lilac aroma, with an odor threshold of 7.2 ppm [65]; 4-ethylphenol (8.4%); 2-amino benzaldehyde (6.7%); benzeneacetic acid methyl ester (5.4%); tetradecanol (4.7%); and hotrienol (3.3%), which has a fresh, sweet, floral aroma and an odor threshold of 0.006 ppm [23].

Notably, hotrienol was the only highly representative compound common to both samples, although present in significantly different proportions (42.4% in *M. interrupta* and 3.3% in *M. seminigra*). This difference highlights the influence of bee species on the chemical composition and sensory properties of honey.

The aromatic characteristics of the major compounds found in *M. interrupta* honey suggest notes of freshness, sweetness, floral and fruity elements, whereas *M. seminigra* honey tends to be sweeter, with almond and fruity notes, according to its chemical profile.

The chemical composition of different honey samples shows great variability, influenced by several factors, including the bee species and the botanical source. This diversity results in honey with distinct chemical characteristics, physical properties, and sensory attributes, reflecting the complexity and uniqueness of each sample [66].

In addition, bees can produce or transform plant-derived constituents into new volatile compounds. During the production of floral honey, the collected nectar is transformed, stored, and matured within the honeycombs, resulting in chemical modifications that influence its volatile profile [67].

These volatile compounds can also be affected by post-harvest processing, including the action of microorganisms present in the environment, which may alter their chemical composition [62,68]. The presence of linalool and its derivatives is commonly reported in honey samples. Reference [69] investigated the relationship between *Melipona* sp. honey and its floral sources and found linalool to be present in all samples analyzed. Other studies have also demonstrated that linalool derivatives originate from the flowers visited by bees [70].

Similarly, when analyzing honey produced by stingless bees, including *M. bicolor*, *M. marginata*, *M. scutellaris*, and *M. quadrifasciata*, it was observed that linalool-derived peaks were the most abundant, regardless of geographical origin or bee species [62].

When evaluating the volatile composition of honey produced by *M. subnitida* and *M. scutellaris* in the semi-arid region of Brazil, the presence of (*Z*)-linalool oxide, hotrienol, rose oxide, nerol oxide, and *α*-terpineol was also identified—compounds that were also detected in the present study. This similarity in chemical composition suggests potential common patterns in the biosynthesis of volatile compounds in stingless bee honey, reinforcing the influence of ecological and botanical factors on the formation of the aromatic profile of these products [71].

Additionally, in honey produced by *M. scutellaris* in the state of Paraíba, the presence of phenethyl alcohol, 1-nonanol, nerol oxide, and α-terpineol was also reported [63]. The compounds (*Z*)-linalool oxide, hotrienol, nerol oxide, and α-terpineol have also been found in honey produced by *M. subnitida*, *M. bicolor*, *M. marginata*, *M. scutellaris*, and *M. quadrifasciata* [62].

The presence of compounds such as hotrienol, linalool, (*Z*)-linalool oxide, nerol oxide, and *α*-terpineol in various samples, despite variations in their concentrations, suggests that these volatiles are frequently present in the aromatic profile of honey within the *Melipona* genus. Future studies may further explore the relationship between botanical origin and the volatile composition of honey, as well as investigate how management and processing practices influence the quality and properties of these honey samples, thereby expanding current knowledge on this product.

Among the major floral compounds, phenethyl alcohol detected in *M. seminigra* honey at 12.3% was also identified in significant proportions in the flowers of *Averrhoa carambola* (1.6%) and *Senna occidentalis* (13.5%). In addition, 1-nonanol, which accounted for 8.08% of the volatile composition in *M. interrupta* honey, was also one of the predominant constituents in the floral profile of *Averrhoa carambola* (1.8%).

These correspondences highlight the importance of ecological interactions between bees and plants. It is worth noting that honey composition is highly variable and influenced by several factors that directly affect its quality and profile, such as bee species, floral origin, environmental conditions, and storage practices [72].

Thus, these findings suggest a correlation between volatile compounds emitted by flowers and the chemical profiles of stingless bee honey. The consistent presence of terpenes, alcohols, and esters, both in floral samples and honey, reinforces the ecological relevance of the local flora and its role in determining the sensory and functional properties of these products [62]. However, when analyzing thirteen honey samples and their respective botanical origins, it was concluded that no single volatile compound could be used as a common marker between the honey and its corresponding flower [65].

The integrated analysis of the obtained data allows for inferring relevant correlations between the chemical composition of honey and the floral sources near the hives, taking into account the limited foraging range of *Melipona* species. Thus, variation in the volatile profile among samples may be associated with the local floristic composition [73]. This botanical diversity can directly influence the presence of bioactive secondary metabolites in honey, also reflected in phenolic compound contents and antioxidant activity [49].

The integrated analysis of the obtained data allows for inferring relevant correlations between the chemical composition of honey and the floral sources near the hives, taking into account the limited foraging range of *Melipona* species. Thus, variation in the volatile profile among samples may be associated with the local floristic composition [20]. This botanical diversity can directly influence the presence of bioactive secondary metabolites in honey, also reflected in phenolic compound contents and antioxidant activity [50].

The integration of chemical data and geobotanical information proves to be a promising tool for the traceability and valorization of regional honey from stingless bees in Amazonian “terra firme” environments.

In addition to identifying volatile compounds such as terpenes and esters in the analyzed honey, it is important to consider their possible formation mechanisms and ecological functions. Terpenes, for example, are synthesized by plants mainly through the mevalonic acid (MVA) and methylerythritol phosphate (MEP) pathways and are widely emitted by flowers as part of their chemical signature. These compounds play essential roles in mediating plant–insect interactions, acting as specific attractants for pollinators or as repellents for herbivores [20].

Volatile esters, in turn, are also common in flowers and fruits, resulting from the esterification between alcohols and organic acids. They contribute to a floral and fruity aroma and play a crucial role in the chemical communication between plants and insects [20].

In the context of meliponiculture, the presence of these volatiles in honey may directly reflect the floral spectrum visited by the bees, functioning as chemical markers of botanical origin. Moreover, these substances can modulate bee behavior, influencing nectar collection, floral selection, and even aspects of colony organization [74].

Therefore, the volatile profile of stingless bee honey not only contributes to its sensory characteristics but also reveals underlying ecological interactions between plants and pollinators. A deeper understanding of these relationships can enhance the recognition of the ecological and functional value of stingless bee honey, especially in complex tropical ecosystems such as the Amazon.

Future studies may further explore these relationships, contributing to the valorization of honey produced by native bees and the conservation of plant species that support their production, ultimately promoting the sustainable development of this productive chain.

## 4. Materials and Methods

### 4.1. Chemicals

The materials used included culture media, bacterial strains, commercial antibiotics, and analytical-grade reagents. Mueller–Hinton broth was purchased from Kasvi (São José dos Pinhais, PR, Brazil). Microorganism strains were obtained from Cefar (São Paulo, SP, Brazil). Levofloxacin was provided by EMS (São Paulo, SP, Brazil). Dextrose, sodium chloride (NaCl), and formic acid were acquired from Dinâmica (São Paulo, SP, Brazil). Resazurin, 2,2-diphenyl-1-picrylhydrazyl (DPPH), 2,2′-azino-bis(3-ethylbenzothiazoline-6-sulfonic acid) (ABTS), potassium persulfate, and Trolox were obtained from Sigma-Aldrich (St. Louis, MO, USA). Methanol was purchased from Qhemis (Barueri, SP, Brazil), and acetonitrile from J.T. Baker (Phillipsburg, NJ, USA). Dimethyl sulfoxide (DMSO) was acquired from Cromato Produtos Químicos (São Paulo, SP, Brazil), and Milli-Q deionized water was obtained from Merck, Darmstadt, Germany).

### 4.2. Collection of Honey and Plant Material Samples

Two honey samples were collected from the species *Melipona interrupta* (MIH) and *Melipona seminigra* (MSH), obtained from a private property, Vivenda do Mel, located in the municipality of Iranduba, Amazonas, Brazil, on 15 August 2023, during the region’s dry season. The honey was collected directly from the hives using sterile disposable syringes. The samples were then transferred to plastic containers and stored at 4 °C ± 2 and protected from light until analysis.

The bee species were identified by MSc. José Augusto dos Santos Silva from the Bee Research Group at the National Institute for Amazonian Research (INPA).

In addition, samples of plant species located near the hives were collected. The identification of the plant material was carried out under the supervision of Ma. Deisy Pereira Saraiva, a biologist at the herbarium of the Federal University of the State of Amazonas. The collected materials are deposited and referenced according to the codes listed in Table 5. All the samples were registered at the National Management System for Genetic Heritage and Associated Traditional Knowledge under the code AAB02D6.

### 4.3. Nutritional Aspects

The honey samples were analyzed according to the methodology described in the Analytical Standards of the Adolfo Lutz Institute [74]. All results were expressed on a fresh weight basis. Moisture content (g/100 g) was determined by the mass difference after drying the sample in an oven at 105 °C. Ash content (g/100 g) was determined by mass loss after incineration in a muffle furnace at 550 °C. Protein content (g/100 g) was estimated based on the total nitrogen content of the sample, measured using the classical Kjeldahl method and calculated using the nitrogen-to-protein conversion factor of 6.25. Lipid content (g/100 g) was determined by direct extraction in a Soxhlet apparatus using *n*-hexane as the solvent. Carbohydrate content (g/100 g) was evaluated by theoretical calculation (by difference), according to the formula: % Carbohydrates = 100 − (% moisture + % protein + % lipids + % ash). Reducing and non-reducing sugars were quantified using Fehling’s reagent. The total caloric value (kcal) was calculated using the following conversion factors: lipids (8.93 kcal/g), proteins (4.27 kcal/g), and carbohydrates (3.82 kcal/g) [75].

### 4.4. Preparation of Extracts

To obtain an extract from each sample, 5 g of honey was solubilized in 50 mL of deionized water and vortexed until complete homogenization. The samples were then subjected to a cleanup procedure using solid-phase extraction (SPE). For this purpose, Strata-X polymeric reversed-phase cartridges (500 mg) (Phenomenex, Torrance, CA, USA) were used. The sample was loaded onto the cartridge, followed by a washing step with 6 mL of deionized water. Subsequently, the compounds were eluted using 10 mL of a methanol:acetonitrile solution (2:1, *v*/*v*). The eluates were collected and placed in a desiccator; after drying, the extracts were stored in a freezer until analysis.

### 4.5. Radical Scavenging Activity

The radical scavenging activity assays were performed according to previously described methods based on the DPPH [76] and ABTS [77] radicals. A Spectramax Plus 384 spectrophotometer (Molecular Devices, San Jose, CA, USA) was used for all colorimetric assays, operating at wavelengths of 517 nm (DPPH) and 734 nm (ABTS). For both assays, the results were expressed as a percentage (%) of radical inhibition and as the half-maximal inhibitory concentration (IC_50_), in μg/mL of crude extract. Trolox was used as a positive control in both assays.

### 4.6. Antibacterial Activity

The microdilution technique [78,79] was used by reducing resazurin (7-hydroxy-3*H*-phenoxazin-3-one-10-oxide) for antibacterial tests and TTC (2,3,5-triphenyltetrazolium chloride) for antifungal tests. All tests were performed in triplicate. The samples were tested against strains purchased commercially from Cefar Diagnóstica Ltd.a*: Escherichia coli* (CCCD-E005), *Staphylococcus aureus* (CCCD-S009), *Klebsiella pneumoniae* (CCCD-K003) *Pseudomonas aeruginosa* (CCCD-P004), *Proteus mirabilis* (CCCD-P001), *Staphylococcus epidermidis* (CCCD-S010), *Enterococcus faecalis* (CCCD-E002), *Salmonella enterica* (CCCD-S003), *Serratia marcescens* (CCCD-S005), *Bacillus subtilis* (CCCD-B005), *Candida albicans* (CCCD-CC001), *Candida tropicalis* (CCCD-CC002), *Candida parapsilosis* (CCCD-CC004). The assay was performed in 96-well microplates with 100 μL of double-concentrated microbial inoculum (5 × 10^5^ CFU/mL—bacteria and 5 × 10^3^ CFU/mL—fungi) and 100 μL of the sample to be tested. The positive control used for bacteria was levofloxacin 0.25 mg/mL, and terbinafine 0.4 mg/mL for fungi. As a negative control, 100 μL of the microbial inoculum and 100 μL of 10% DMSO were inserted. For sterility control, 100 µL of the sterile culture medium used to prepare the inoculum [Mueller Hinton broth for bacteria (beef extract—2.0 g/L; casamino acid—17.5 g/L; starch—1.5 g/L) and Sabouraud broth for fungi (dextrose—20 g/L; peptone—10g/L)] was added.

The microplates were then incubated at 37 °C for 24 h (bacteria) and 48 h (fungi) in a BOD incubator. After this period, 30 µL of resazurin was added to all wells of the antibacterial activity assay plates, and 30 µL of TTC was added to the wells of the antifungal activity assay plates. The plates were incubated again at 37 °C for 1 to 2 h to check the reduction of the inserted developers. For the antibacterial assay, the pink color is indicative of cell growth, i.e., no antimicrobial action, and for the antifungal assay, the red color indicates that there was no inhibition of microbial growth by the extract tested.

For extracts with antimicrobial activity, the Minimum Inhibitory Concentration (MIC) was determined by successive dilutions of the samples with sterile distilled water. The concentration ranges used were 75, 37.5, 18.7, 9.4, 4.7, and 2.3 µg/mL, where the lowest concentration of extract that managed to inhibit microbial growth was considered the MIC.

### 4.7. Analysis of Volatile Organic Compounds (VOCs)

The composition of volatile organic compounds (VOCs) was investigated using headspace solid-phase microextraction coupled with gas chromatography–mass spectrometry (HS-SPME/GC–MS). Honey samples (approximately 2.00 g) were weighed into 10 mL SPME vials, followed by the addition of 2 mL of distilled water and 200 mg of sodium chloride (NaCl) (100 mg/mL ~ 1.71 mol/L). A pre-extraction incubation was carried out under agitation for 30 min at 40 °C. VOC extraction was performed using an SPME fiber assembly coated with divinylbenzene/carboxen/polydimethylsiloxane (DVB/CAR/PDMS) (Sigma-Aldrich, St. Louis, MO, USA) for 60 min in the headspace. After extraction, the fiber was desorbed in the GC injector for 5 min at 270 °C.

Untargeted analyses were performed using a GC-MS system (Shimadzu QP2010 Plus, Kyoto, Japan) consisting of a gas chromatograph coupled to a quadrupole mass spectrometer equipped with an electron ionization (EI) source at 70 eV. Chromatographic separation was achieved using an Rtx-5MS^®^ capillary column (5% phenyl and 95% methylpolysiloxane) (Sulpeco, Bellefonte, PA, USA) (30 m × 0.25 mm i.d., 0.25 μm film thickness) with helium as the carrier gas at a flow rate of 1.0 mL/min. The oven temperature program was as follows: initial temperature of 50 °C (held for 1 min), increased at 3 °C/min to 270 °C (held for 1 min), then increased at 5 °C/min to 300 °C (held for 10 min). The injector operated in splitless mode, with helium (99.99% purity) as the carrier gas at a flow rate of 1.2 mL/min.

The proportion of each compound was estimated by dividing its average peak area by the total average chromatographic area and expressed as relative percentages. Compound identification was based on similarity with entries in the NIST library, elution order, and retention indices (RI). A mixture of linear alkanes (C_7_–C_30_) was analyzed alongside the samples to calculate RI values according to the van den Dool–Kratz equation [80].

### 4.8. Statistical Analysis

All tests were performed in triplicate. Mean values for different species were compared by one-way ANOVA, considering differences significant when *p* < 0.05.

## 5. Conclusions

The chemical profile of the analyzed samples was predominantly composed of terpenes, with linalool standing out as a major compound. Linalool is a monoterpene widely distributed in plant species of medicinal interest. The presence of these volatile compounds reflects the close ecological relationship between *M. interrupta* and *M. seminigra* bees and the local flora, as a portion of the secondary metabolites detected in the honey originates from the plants visited during foraging. The physicochemical parameters observed are consistent with data reported in the literature for *Melipona* honey, reinforcing the nutraceutical potential of honey produced by these species. The antibacterial activity observed against *E. faecalis* and *B. subtilis* suggests possible synergistic effects between plant-derived metabolites and enzymatic components introduced by the bees during nectar processing. The antioxidant activity detected in the samples may be associated with the presence of phenolic and terpenoid compounds, contributing to the functional properties of these honey samples. These findings underscore not only the ecological importance of plant–pollinator interactions in the transfer of bioactive compounds but also highlight the still underexplored potential of meliponiculture products as functional foods. Furthermore, they provide scientific support for the traditional use of honey produced in Iranduba, Amazonas (Brazil).

## Figures and Tables

**Table 1:** Physicochemical parameters in honey samples of *M. interrupta* and *M. seminigra*.

Parameter	MIH (*M. interrupta*)	MSH (*M. seminigra*)
Moisture (g/100 g)	20.11 ± 0.61	27.10 ± 0.13
Ash (g/100 g)	0.15 ± 0.06	0.31 ± 0.05
Protein (g/100 g)	0.19 ± 0.09	0.16 ± 0.01
Lipid (g/100 g)	0.20 ± 0.12	0.19 ± 0.08
Carbohydrate (g/100 g)	79.35 ± 0.62	72.24 ± 0.32
Reducing sugar (%)	76.95 ± 0.51	68.75 ± 0.17
Non-reducing sugar (%)	1.43 ± 0.69	3.14 ± 0.13
Total caloric value (kcal/100 g)	305.71 ± 2.34	278.34 ± 0.52

**Table 2:** Radical scavenging activity of *M. seminigra* and *M. interrupta*.

	Radical Scavenging Activity			
Samples	DPPH		ABTS	
MIH	987.74 ± 0.02 ^a^	49.98 ± 0.75 ^b^	65.49 ± 0.02 ^a^	58.73 ± 2.33 ^b^
MSH	953.31 ± 0.01 ^a^	46.14 ± 1.83 ^b^	67.73 ± 0.01 ^a^	60.17 ± 1.68 ^b^

^a^: IC_50_ expressed in μg/mL, ^b^: % of radical reduction, expressed as %.

**Table 3:** Antimicrobial activity of *M. seminigra* and *M. interrupta*.

Samples	EC	SA	PA	PM	BS	SE	EF	SM	KP	SE	CA	CT	CP
MIH	-	-	-	-	75	-	75	-	-	-	-	-	-
MSH	-	-	-	-	75	-	75	-	-	-	-	-	-

Minimum Inhibitory Concentration (MIC) of the tested samples expressed in µg/mL. **EC**—Escherichia coli; **SA**—Staphylococcus aureus; **PA**—Pseudomonas aeruginosa; **PM**—Proteus mirabilis; **BS**—Bacillus subtilis; **SE**—Staphylococcus epidermidis; **EF**—Enterococcus faecalis; **SM**—Serratia marcescens; **KP**—Klebsiela pneumoniae; **SE**—Salmonella entérica; **CA**—Candida albicans; **CT**—Candida tropicalis; **CP**—Candida parapsilosis; - No activity.

**Table 4:** Volatile organic compounds identified in honey and flower samples.

Compounds	M/W	RT	RI_experimental_	RI_theoretical_	Relative Area (%)						
					MIH	MSH	AO	MI	AC	BO	SO
(3*Z*)-Hexen-1-ol	101	6.36	846	858					7.43	7.14	
α-Pinene	136	8.76	924	932				19.04			
α-Thujene	136	8.77	934	924						1.43	
Benzaldehyde	106	9.89	956	960		14.68					
Sabinene	136	10.53	966	969				2.95			
β-Pinene	136	10.70	971	974				8.64			
1-Octen-3-ol	128	10.85	982	986					0.73		
β-Myrcene	136	11.15	984	988				10.43			
3-Carene	136	11.66	1005	1008			6.12	20.91			
2-Ethyl-1-hexanol	130	12.32	1025	1029	2.19						
D-Limonene	136	12.38	1053	1033				6.92			
(*Z*)-β-Ocimene	136	12.59	1033	1050					3.14		0.50
Eucalyptol	154	12.65	1034	1031						1.26	
Benzyl alcohol	108	12.79	1038	1034	0.89		0.89				
Benzene acetalheyde	120	12.83	1039	1042		1.00					
(*E*)-β-Ocimene	136	12.96	1045	1050	0.45				50.93	8.80	40.68
γ-Terpinene	136	13.39	1112	1064				2.35			
(*Z*)-Linalool oxide	170	13.77	1067	1067	9.20					1.06	
Terpinolene	136	14.45	1068	1088		1.11	1.01	11.49			
(*E*)-Linalool oxide	213	14.51	1089	1080					0.41	14.30	
*p*-Cymene	134	14.62	1184	1021				2.23			
*p*-Cymenene	132	14.64	1093	1089			0.21				
Linalool	154	14.68	1094	1098	2.65	1.61	0.12	3.86	1.26	1.59	
Hotrienol	152	14.86	1099	1101	42.42	3.33					
Rose oxide	154	15.05	1105	1112	2.15						
Nonanal	142	15.11	1107	1102			0.10		5.43		
4.8-Dimethylnona 1.3.7-triene	150	15.26	1114	1110				5.71			
Phenethyl alcohol	122	15.39	1116	1114		12.27	1.26		1.63		13.47
(*E*)-*p*-Mentha-1(7).8-dien-2-ol	152	15.64	1124	1185	1.66						
*neo*-*allo*-Ocimene	136	15.84	1130	1131					1.31		7.57
Cosmene	134	15.90	1132	1130					1.83	4.78	5.17
Hexyl isobutyrate	172	16.26	1144	1150						3.45	
Benzyl nitrile	117	16.30	1145	1143			3.49				
Nerol oxide	152	16.42	1149	1151	7.23						
4-Ethylphenol	122	16.87	1163	1163		8.46					
*p*-Mentha-1.5-dien-8-ol	152	16.89	1164	1159	4.95						
1-Nonanol	144	16.92	1165	1169	8.08		0.12		1.77		
Lavandulol	154	16.97	1167	1161					2.44		
Octanoic acid	144	17.16	1173	1191		1.83					
(*E*)-Linalool-3.7.oxide	170	17.34	1178	1178						0.61	
α-Terpineol	154	17.57	1186	1189	7.36					0.15	
Ethyl caprylate	172	17.72	1191	1197		1.67					
Methyl salicylate	152	17.93	1197	1187			2.38				1.05
Decanal	156	18.07	1198	1200	0.62	0.02				0.33	
2-Amino benzaldehyde	121	18.33	1211	1222		6.68					
3-Hexenyl 2-methylbutyrate	184	19.08	1264	1233						26.35	0.13
Isovaleric acid-(*Z*)-3-hexenyl ester	184	19.09	1237	1238							0.12
Benzeneacetic acid methyl ester	491	19.14	1239	1234		5.39					
β-Phenethyl acetate	164	19.70	1251	1245		0.46					0.12
Nonanoic acid	158	19.88	1262	1272	0.57	2.41					
Hydroquinone	110	20.03	1269	1241		1.12					
(*E*)-Cinnamaldehyde	132	20.34	1280	1266			0.64				
Nonanoic acid ethyl ester	186	20.54	1287	1294		1.49					
Cinnamyl alcohol	134	21.26	1313	1312			0.64				
Decanoic acid methyl ester	186	21.36	1317	1328		2.58					
α-Cubebene	204	22.29	1351	1351			1.06				0.50
Decanoic acid	172	22.53	1360	1364		2.41					
Ylangene	204	22.92	1375	1373			1.88				0.36
α-Copaene	204	23.13	1383	1372			6.68				1.40
Capric acid	172	23.18	1385	1381		0.17					
β-Bourbonene	204	23.29	1388	1384							0.24
(*Z*)-Jasmone	164	23.34	1390	1392	2.56						
Sativene	204	23,58	1399	1390			0.19				
β-Longipinene	204	23.66	1404	1400			0.15				
Dodecanal	184	23.71	1412	1412						0.88	
β-Caryophyllene	204	24.28	1427	1418			26.22				12.31
α,β-Dihydro-β-ionone	194	24.56	1436	1438							1.90
Nerylacetone	194	24.63	1441	1445	0.59						
α-Bergamotene	204	24.80	1438	1432			3.13				
α-Guaiene	204	25.56	1445	1440							0.60
Aromandendrene	204	28.89	1447	1436			2.07				
1-Dodecanol	186	25.17	1462	1469		2.82					
γ-Gurjunene	204	25.25	1465	1479			4.69				0.37
α-Caryophyllene	204	25.26	1466	1455			5.13				
γ-Muurolene	204	25.72	1484	1477			4.08				3.18
α-Amorphene	204	25.80	1487	1484			0.33				0.38
α-Farnesene	204	26.31	1507	1507					16.63		0.12
β-Bisabolene	204	26.47	1513	1506			1.95				
γ-Cadinene	204	26.68	1523	1513			4.72				
δ-Cadinene	204	26.82	1529	1523			5.00				
(*Z*)-Calamene	202	26.90	1532	1523			2.46				
α-Calacorene	200	27.30	1549	1546							0.41
Cadine-1.4-diene	204	27.38	1542	1539			1.84				
Dodecanoic acid	200	27.34	1565	1566		1.66					
Selina-3.7(11)-diene	204	27.39	1552	1545			3.30				
Caryophylene oxide	220	27.70	1560	1578			0.26				
(3*E*,7*E*)-4.8.12-Trimethyltrideca-1.3.7.11-tetraene	218	27.94	1576	1580							6.84
3-Hexen-1-ol-benzoate	204	28.00	1579	1565			1.95			0.52	
Tetradecanol	214	29.86	1660	1671		4.72					
Hexadecanol	242	34.12	1866	1875		0.41					
					93.57	85.45	86.97	94.53	94.95	72.65	97.49

RI_exp_: experimental retention index, RI_the:_ teorical retention index.

**Table 5:** Identification of deposits corresponding to plant species collected in Iranduba—AM.

Sample	Sample ID	Deposit Code	Latitude	Longitude
*Anacardium occidentale*	AO	HUAM 12685	3°16′2.7″	60°11′28.3″
*Mangifera indica*	MI	HUAM 12686	3°16′2.4″	60°11′29.0″
*Averrhoa carambola*	AC	HUAM 12687	3°16′2.9″	60°11′28.8″
*Bougainvillea* sp.	BO	HUAM 12688	3°16′3.0″	60°11′27.7″
*Senna occidentalis*	SO	HUAM 12689	3°16′2.7″	60°11′27.7″

## Data Availability

Data are contained within the article.

## Supplementary Materials

The following supporting information can be downloaded at: https://www.mdpi.com/article/10.3390/plants14142106/s1.

## Abbreviations

ABTS	Chemical compound 2,2′-azino-bis(3-ethylbenzothiazoline-6-sulfonic acid)
AC	Flowers of *Averrhoa carambola*
AM	Amazonas state
AO	Flowers of *Anacardium occidentale*
BO	Flowers of *Bougainville* sp.
BOD	Biochemical Oxygen Demand
CLSI	Clinical and Laboratory Standards Institute
DMSO	Dimethyl sulfoxide
DPPH	Organic chemical compound 2,2-diphenyl-1-picrylhydrazyl
DVB/CAR/PDMS	Divinylbenzene/carboxen/polydimethylsiloxane
EI	Electron Ionization
GC	Gas Chromatography
GC-MS	Gas Chromatography Coupled to Mass spectrometry
HOMA-β	Homeostasis Model Assessment of β-cell Function
HS-SPME/GC–MS	Headspace Solid-phase Microextraction Coupled with Gas Chromatography–Mass Spectrometry
IC_50_	Inhibitory Concentration 50%
INPA	National Institute for Amazonian Research
LPS	Lipopolysaccharides
MI	Flowers of *Mangifera indica*
MIC	Minimum Inhibitory Concentration
MIH	Honey of *Melipona interrupta*
MSH	Honey of *Melipona seminigra*
RI	Retention Indices
SO	Flowers of *Senna occidentalis*
SPE	Solid-Phase Extraction
SPME	Solid-Phase Microextraction
UEA	University of the State of Amazonas
UFAM	Federal University of Amazonas
VOC	Volatile Organic Compound
VOCs	Volatile Organic Compounds
