# Supplementary material for: Nutritional Composition, Volatile Profiles, and Biological Evaluation of Honeys from Melipona interrupta and Melipona seminigra from Amazonas State, Brazil
Source: Plants (Basel). 2025 Jul 9;14(14):2106. doi: 10.3390/plants14142106 (PMC12299362; doi:10.3390/plants14142106)
Supplement: Supplementary file 1 [file plants-14-02106-s001.zip › plants-3645633-supplementary.pdf]

SUPPLEMENTARY MATERIAL

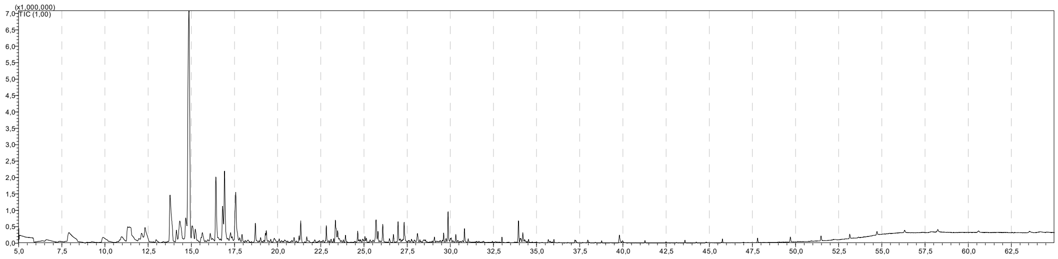

Figure S1. chromatogram of VOCs in MIH sample

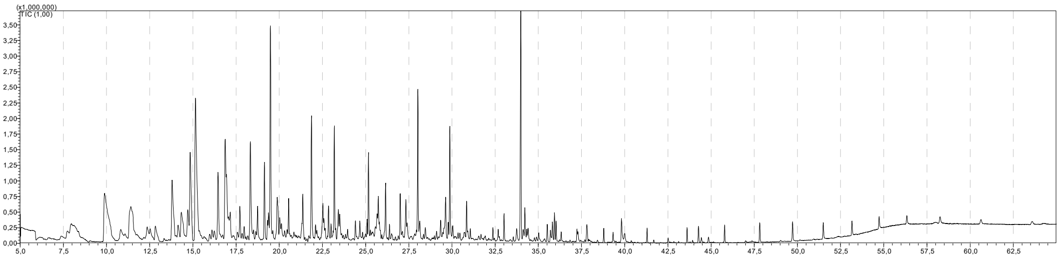

Figure S2. chromatogram of VOCs in MSH sample

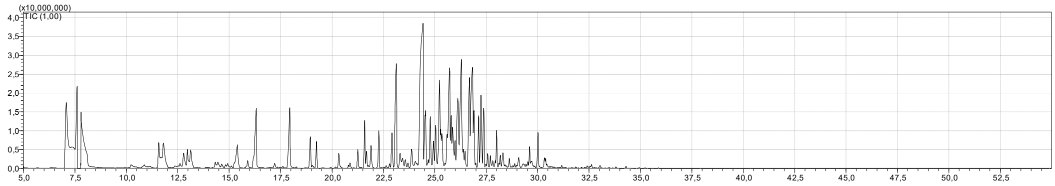

Figure S3. chromatogram of VOCs in AO sample

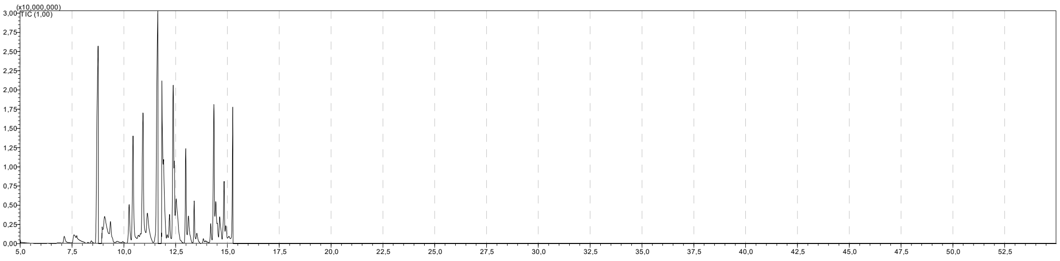

Figure S4. chromatogram of VOCs in MI sample

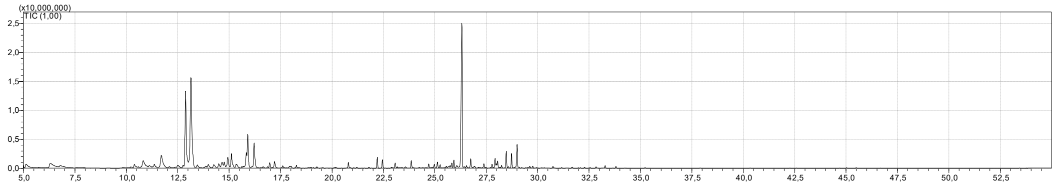

Figure S5. chromatogram of VOCs in AC sample

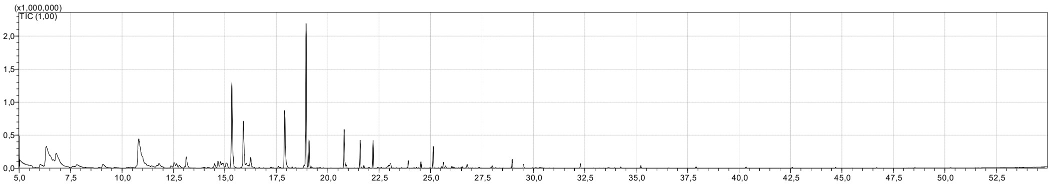

Figure S6. chromatogram of VOCs in BO sample

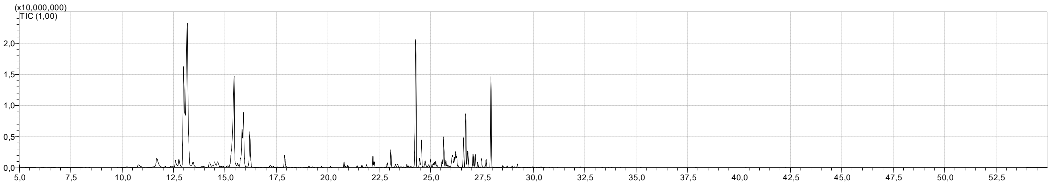

Figure S7. chromatogram of VOCs in SO sample
